# Predictive factors of needle-knife pre-cut papillotomy failure in patients with difficult biliary cannulation

**DOI:** 10.1038/s41598-022-09117-9

**Published:** 2022-03-23

**Authors:** Mu-Hsien Lee, Shu-Wei Huang, Cheng-Hui Lin, Yung-Kuan Tsou, Kai-Feng Sung, Chi-Huan Wu, Nai-Jen Liu

**Affiliations:** Department of Gastroenterology and Hepatology, Chang Gung Memorial Hospital and Chang Gung University College of Medicine, 5, Fu-Shin Street, Kweishan, Taoyuan 333, Taiwan, ROC

**Keywords:** Gastrointestinal diseases, Gastrointestinal system

## Abstract

Predictors of needle-knife pre-cut papillotomy (NKP) failure for patients with difficult biliary cannulation has not been reported. Between 2004 and 2016, 390 patients with difficult biliary cannulation undergoing NKP were included in this single-center study. Following NKP, deep biliary cannulation failed in 95 patients (24.4%, NKP-failure group) and succeeded in 295 patients (75.6%, NKP-success group). Patient and technique factors were used to identify the predictors of initial NKP failure. Compared with the NKP-success group, periampullary diverticulum (28.4% vs. 18%, p = 0.028), surgically altered anatomy (13.7% vs. 7.1%, p = 0.049), number of cases performed by less experienced endoscopists, and bleeding during NKP (38.9% vs. 3.4%, p < 0.001), were significantly more frequent in the NKP-failure group. On multivariate analysis, surgically altered anatomy (OR 2.374, p = 0.045), endoscopists’ experience (OR 3.593, p = 0.001), and bleeding during NKP (OR 21.18, p < 0.001) were significantly associated with initial failure of NKP. In conclusion, NKP is a highly technique-sensitive procedure, as endoscopists’ experience, bleeding during NKP, and surgically altered anatomy were predictors of initial NKP failure.

## Introduction

Endoscopic retrograde cholangiopancreatography (ERCP) has been considered as the most important therapeutic procedure for biliary disease^1^. Deep bile duct cannulation is a key step in the success of therapeutic ERCP, but it cannot be always achieved^2^. It can be difficult or even impossible in approximately 5–20% of patients by using conventional cannulation methods^3^. Patients with such difficulties generally have papillary stenosis, impacted stones in the distal end of the common bile duct (CBD), a displaced papillary orifice caused by periampullary tumor or diverticulum, abnormal papillary position, or surgically altered anatomy^4–7^. In such cases, pre-cut techniques are most often used as a salvage procedure after failure of conventional methods of bile duct cannulation^8,9^. Needle-knife pre-cut sphincterotomy, including needle-knife pre-cut papillotomy (NKP) and needle-knife fistulotomy (NKF), is probably the most widely used pre-cut technique^9–11^. NKP is preferred by some centers or some endoscopists. However, mastering the skills required for NKP is difficult^12^. On literature search, the reported initial success rate of NKP in difficult cannulation cases ranged widely from 71.3 to 92%^10,13–19^. Therefore, some factors may contribute to the initial failure of NKP, on which there are no reported studies. We conducted this study to analyze the factors associated with the initial failure of NKP in patients with difficult bile duct cannulation.

## Materials and methods

Between January 2004 and December 2016, a total of 8979 patients with an intact major papilla who underwent ERCP due to biliary tract diseases were selected from the database of the Therapeutic Endoscopic Center of Chang Gung Memorial Hospital, Linkou Medical Center, Taoyuan, Taiwan. Among them, 469 (5.2%) patients underwent needle knife pre-cut sphincterotomy. Patients who had an indwelling biliary stent (n = 34) or a nasobiliary drainage tube (n = 5) before pre-cut sphincterotomy, or who had impacted stones in the ampulla of Vater receiving pre-cut sphincterotomy without an attempt of conventional cannulation methods (n = 33), were excluded from the study. Therefore, 397 patients who met the definition of difficult biliary cannulation (as described below) and underwent pre-cut sphincterotomy were enrolled. Because the patient number was small, seven patients who underwent NKF were further excluded in the study. The study was approved by the institutional review board of Chang Gung Memorial Hospital (202000751B0C601). Since this is a retrospective study using clinical routine treatment or diagnostic medical records, and no human immunodeficiency virus-positive cases were involved, the Chang Gung Medical Foundation Institutional Review Board approved the waiver of the participant's consent. All methods were carried out in accordance with relevant guidelines and regulations.

### ERCP and NKP procedures

All patients underwent ERCP in the prone position and intravenous sedation with midazolam and fentanyl (meperidine in the early period). The duodenoscopes used were either TJF-240/260 or JF-240/260 (Olympus Optical Co. Ltd, Tokyo, Japan). All procedures were performed by five endoscopists (A–E). The annual mean number of ERCP cases for endoscopists A, B, C, D, and E was 300, 200, 200, 200, and 130, respectively. Only endoscopist A (performing ERCP since 1995) had NKP experience before the study. Because endoscopists B, C, D, and E learned the ERCP/NKP techniques of endoscopist A, there was no difference in the choice of initial cannulation method among endoscopists. Initial selective cannulation of the CBD was attempted with a cannula or pull-type sphincterotome, depending on the preference of the individual endoscopist. Contrast-assisted cannulation was performed in the early stages of the study, while wire-guided cannulation was the preferred method in the later stages. Bile duct cannulation using a cannula or sphincterotomy with contrast guidance and guidewire assistance is generally considered the standard cannulation technique^20,21^. When these methods failed, the double guidewire technique was sometimes performed by some of the endoscopists if the pancreatic duct (P-duct) was cannulated. Since the double guidewire technique was not difficult, the endoscopists could perform bile duct cannulation in the usual way (standard technique) after placing the guidewire in the P-duct. Therefore, the standard technique together with the double guide wire technique was considered as the conventional cannulation methods in this study. Difficult bile duct cannulation was defined when the conventional cannulation methods failed to achieve deep bile duct cannulation. In this case, the late pre-cut strategy (cannulation time often exceeded 20 min) was carried out prior to 2015, and early pre-cut strategy was frequently adopted after 2015^8,22^. Early NKP was performed when cannulation time was > 5–10 min or unintentional P-duct cannulation was performed more than once^21,23^.

**Figure 1:**
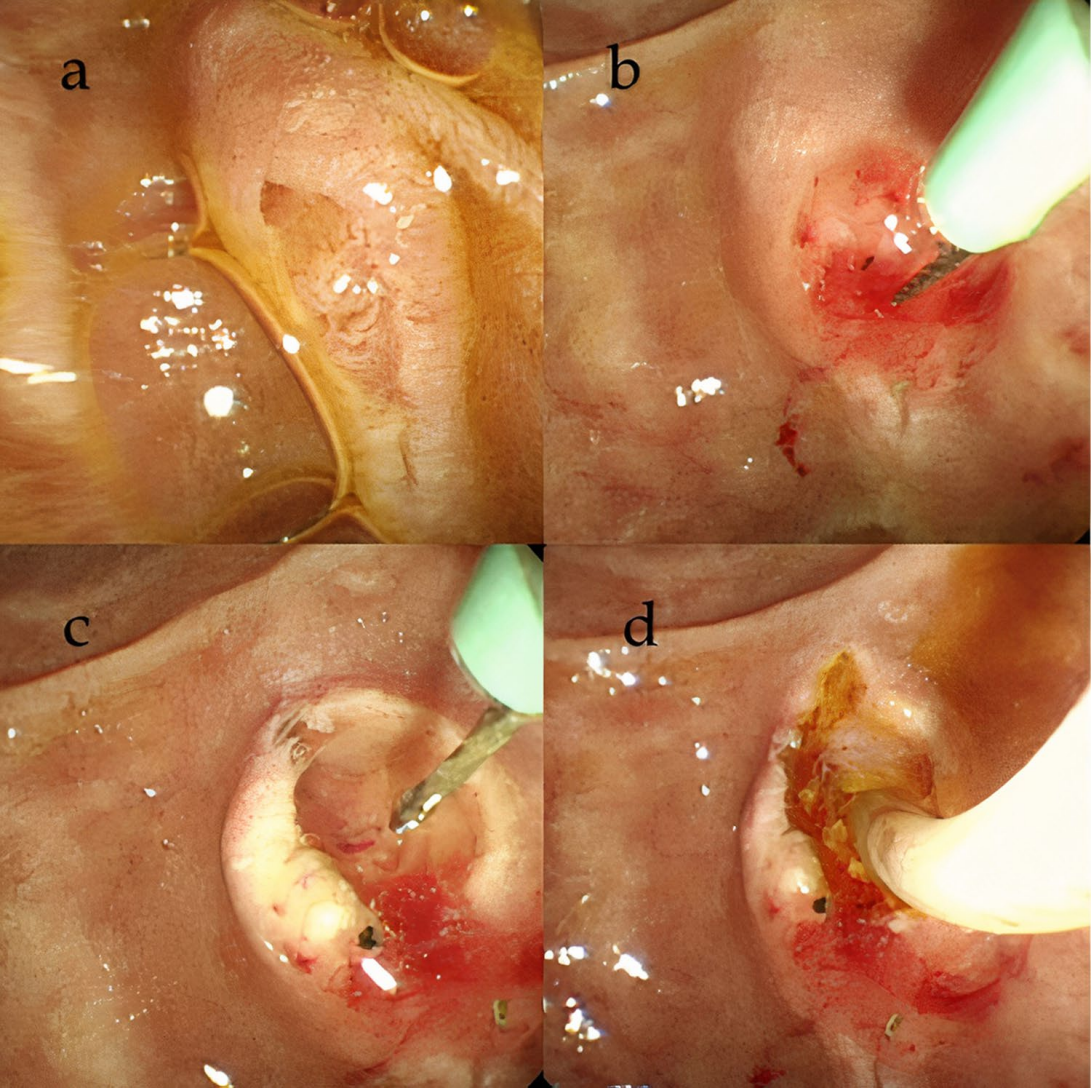
Needle-knife pre-cut papillotomy. (**a**) Original major papilla. (**b**) The incision is made upward along the axis of the bile duct from the papillary orifice. (**c**) The overlying mucosa is incised, and the bile duct is exposed. (**d**) Successful biliary cannulation is achieved.

NKP was performed immediately after the failure of the conventional cannulation methods during the same endoscopic session. The NKP method was the same as that described in our previous study^24^, as shown in Fig. 1. The needle-knife sphincterotome (Rx Needle-Knife XL; Boston Scientific Corporation, Marlborough, USA) and an ICC 200 or VIO 200D electrosurgical unit (ERBE, Tübingen, Germany), which produced blended current, were used for NKP. After puncturing the papilla above the orifice, the incision was made upward along the axis of the bile duct from the papillary orifice. The incision was extended until the CBD was exposed, followed by a small incision in the biliary sphincter muscle. The CBD was then cannulated directly with the closed needle-knife or with a wire-guided cannula/sphincterotome. The success of the NKP procedure was defined as the deep placement of a catheter or a sphincterotome into the CBD with the acquisition of a satisfactory cholangiogram. After achieving deep cannulation, a pull-type sphincterotomy was used to extend it towards the duodenal wall superiorly. Whether to place a P-duct stent before NKP depended on whether the P-duct had been cannulated or as per the judgment of the endoscopist.

### Predictors

**Figure 2:**
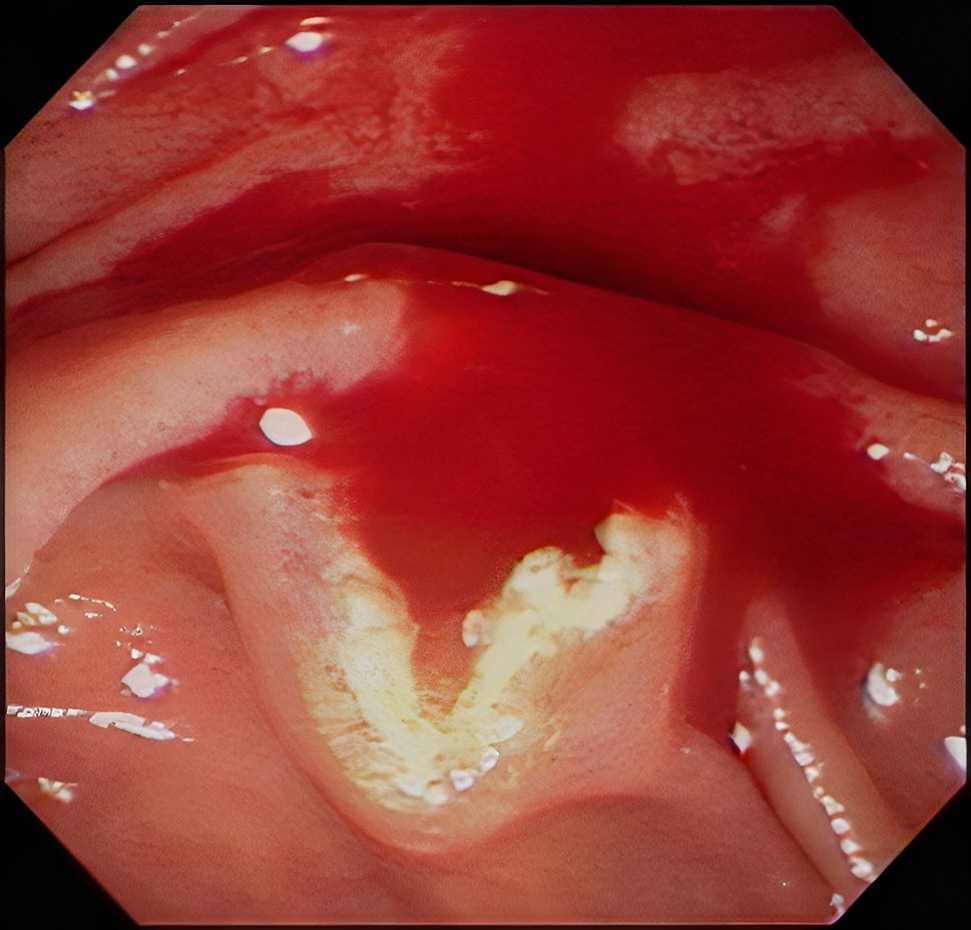
Bleeding during needle-knife pre-cut papillotomy could result in failure of the procedure.

Both patient and technique factors were used to identify the predictors associated with the failure of initial NKP^25^. Patient factors were similar to those of difficult biliary cannulation, including age, sex, the diameter of CBD, major papilla status, and indications of ERCP^4,5^. Major papilla status included periampullary diverticulum, enlarged or swollen papilla, low-set papilla, impacted stone at the ampulla of Vater, periampullary tumor, and surgically altered anatomy. The indications of ERCP included choledocholithiasis, malignant or benign biliary stricture, bile leak, and suspected Sphincter of Oddi dysfunction. Technique factors, based on few individual reports, were decided on consensus by the authors, including the endoscopists’ experience in NKP, bleeding during NKP, early vs. late pre-cut, and placement of a P-duct stent before NKP^25–27^. Bleeding during NKP was defined as bleeding induced by NKP, which could interfere with or eventually cause interruption of NKP (due to blocking of the endoscopic view, Fig. 2)^26^.

### Statistical analysis

Statistical analysis was performed using chi-squared test or Fisher’s exact test (when appropriate) and independent Student’s t-test for categorical and continuous variables, respectively, between the groups of patients with failed and successful NKP. Continuous variables were expressed as median with range. Logistic regression analysis was performed to identify factors associated with initial NKP failure. Statistical analyses were performed using SPSS software (version 20.0; SPSS, Inc., Chicago, IL, USA). A two-tailed p-value of < 0.05 was considered statistically significant.

### Informed consent statement

Due to the retrospective nature of the study, the institutional review board waived the requirement for patient consent.

## Results

The percentage of difficult cannulation requiring NKP was 4% (199/5367) in the contrast-guided cohort and 6.2% (191/3063) in the guidewire-assisted cohort (p = 0.462). During the initial ERCP session, after NKP, deep bile duct cannulation failed in 95 patients (24.4%, NKP-failure group) and was successful in 295 patients (75.6%, NKP-success group). According to the baseline characteristics of patients as listed in Table 1, the median age of 390 patients was 72 years, and the difference was not significant between the two groups. Male patients comprised 52.3%, and the ratio between the two groups was similar. Choledocholithiasis was the most common indication of ERCP (56.4%), followed by malignant biliary stricture (33.6%). There was no significant difference between the two groups on any indication of ERCP. The median CBD diameter was 1.2 cm, which was similar between the two groups. Periampullary diverticulum accounted for 20.5% of the study population, and it was significantly more frequent in the NKP-failure group (28.4% vs. 18.0%, *p* = 0.028). An enlarged or swollen papilla was present in 11.3% of the patients, and the ratios of the two groups were similar. Thirteen patients (3.3%) had impacted CBD stones in the ampulla of Vater, and all were in the NKP-success group (0 vs. 4.4%, *p* = 0.037). The proportions of patients with periampullary tumors and a low-set papilla were 5.4% and 3.6%, respectively, which were not statistically significant between the two groups. Surgically altered anatomy accounted for 8.7% of the study population, and it was significantly more frequent in the NKP-failure group (13.7% vs. 7.1%, *p* = 0.049).

**Table 1:** Baseline characteristics of the patients.

Variables	Total (n = 390)	NKP failure group (n = 95)	NKP success group (n = 295)	*p*-value
Median age, years (range)	72 (21–96)	72 (27–93)	71 (21–96)	0.875
Male, n	204(52.3%)	51(53.7%)	153 (51.9%)	0.757
**Indications of ERCP**				
Choledocholithiasis, n	220(56.4%)	56 (58.9%)	164 (55.6%)	0.566
Malignant biliary stricture, n	131(33.6%)	32 (33.7%)	99 (33.6%)	0.982
Benign biliary stricture, n	23 (5.9%)	4 (4.2%)	19 (6.4%)	0.422
Bile leak, n	14 (3.6%)	2 (2.1%)	12 (4.1%)	0.371
Sphincter of Oddi dysfunction, n	2 (0.5%)	1 (1.1%)	1 (0.3%)	0.397
Median CBD diameter, cm (range)	1.2 (0.4–4.1)	1.2 (0.4–4.1)	1.2 (0.4–2.9)	0.866
**Major papilla status**				
Periampullary diverticulum, n	80 (20.5%)	27 (28.4%)	53 (18.0%)	0.028
Enlarged or swelling, n	44 (11.3%)	12 (12.6%)	32 (10.8%)	0.633
Impacted stone, n	13 (3.3%)	0 (0%)	13 (4.4%)	0.037
Tumor, n	21 (5.4%)	7 (7.4%)	14 (4.7%)	0.325
Low set papilla, n	14 (3.6%)	3 (3.2%)	11 (3.7%)	0.795
Surgically altered anatomy, n	34 (8.7%)	13 (13.7%)	21 (7.1%)	0.049

*CBD* common bile duct, *NKP* needle-knife pre-cut papillotomy, *ERCP* endoscopic retrograde cholangiopancreatography procedure.

Technique factors and NKP-related complications were listed in Table 2. During the study period, endoscopists A, B, C, D, and E performed 186, 93, 49, 43, and 19 NKP procedures, respectively, with success rates of 80.1% (149/186), 76.3% (71/93), 73.5% (36/49), 62.8% (27/43), and 63.2% (12/19), respectively. For statistical analysis, endoscopist A was used as a reference, B and C were considered to have intermediate experience, and D and E together were considered inexperienced. There was a significant difference between the two groups regarding the endoscopists’ experience (*p* = 0.024). The occurrence of bleeding during NKP was significantly more frequent in the NKP-failure group than that in the success group (38.9% vs. 3.4%, *p* < 0.001). A P-duct stent was placed before NKP in 39 (10%) patients. The ratio of P-duct stent placement was not significantly different between the two groups (8.4% vs. 10.5%, p = 0.694). Ninety-six patients underwent early pre-cut and 294 patients underwent late pre-cut. There was no difference in NKP success between the early and late pre-cut groups (74.8% or 75/96 vs. 78.1% or 220/294, p = 0.555). Early pre-cut was used in 22.1% of NKP-failure group and 25.4% in NKP-success group (p = 0.514).

**Table 2:** Technique factors, and adverse events of needle knife pre-cut papillotomy.

Variables	Total (n = 390)	NKP failure group (n = 95)	NKP success group (n = 295)	*p*-value
**Endoscopists, n**				0.024
A	186 (47.7%)	37 (38.9%)	149 (50.5%)	
B + C	142 (36.4%)	35 (36.8%)	107 (36.3%)	
D + E	62 (15.9%)	23 (24.2%)	39 (13.2%)	
Bleeding during NKP, n	47 (12.1%)	37 (38.9%)	10 (3.4%)	< 0.001
P-duct stent	39 (10%)	8 (8.4%)	31 (10.5%)	0.694
Early pre-cut	96 (24.6%)	21 (22.1%)	75 (25.4%)	0.514
**Overall adverse events, n**	27(6.9%)	4 (4.2%)	23 (7.8%)	0.496
Perforation	2 (0.5%)	1 (1.1%)	1 (0.3%)	0.397
Pancreatitis	4 (1.0%)	1 (1.1%)	3 (1.0%)	0.976
Delayed bleeding	13 (3.3%)	1 (1.1%)	12 (4.1%)	0.154
Cholangitis	8 (2.1%)	1 (1.1%)	7 (2.4%)	0.430

*NK*P needle-knife pre-cut papillotomy, *P-duct* pancreatic duct.

Adverse events occurred in 6.9% (27/390) of patients, with no significant difference between the two groups (4.2% vs 7.8%, p = 0.496). Adverse events included perforation (1.1% vs. 0.3%, p = 0.397), pancreatitis (1.1% vs. 1%, p = 0.976), delayed bleeding (1.1% vs. 4.1%, p = 0.154), and cholangitis (1.1% vs. 2.4%, p = 0.43).

In the NPK failure group (n = 95), 34 patients (36.8%) received interval ERCP, 16 patients (16.8%) received bile duct surgery, and 23 patients (24.2%) received percutaneous transhepatic biliary drainage. The remaining 22 patients (23.2%) did not receive any other salvage therapy due to patient refusal or MRCP (performed after initial ERCP) without bile duct lesions. The mean interval between initial and interval ERCPs was 4.3 days (range 1–9 days). A successful interval ERCP was performed in 28 patients (82.4%).

On univariate analysis, the predictive factors of initial NKP failure were periampullary diverticulum, surgically altered anatomy, endoscopists’ experience, and bleeding during NKP (Table 3). On multivariate analysis, surgically altered anatomy (odds ratio [OR] 2.374, 95% confidence interval [CI] 1.020–5.526, *p* = 0.045), endoscopists’ experience (OR 3.593, 95% CI 1.746–7.394, *p* = 0.001 for endoscopists D + E), and bleeding during NKP (OR 21.18, 95% CI: 9.614–46.68, *p* < 0.001) remained statistically significant.

**Table 3:** Univariate and multivariate analyses of the factors associated with the initial failure of needle-knife pre-cut sphincterotomy.

Variables		Univariate analysis		Multivariate analysis	
		OR (95% CI)	*p*-value	OR (95% CI)	*p-*value
Age	> 70 years	1.107 (0.696–1.761)	0.667		
	≤ 70 years	Referent			
Sex	Female	0.930 (0.585–1.478)	0.757		
	Male	Referent			
Choledocholithiasis	Yes	1.147 (0.718–1.833)	0.567		
	No	Referent			
Malignant biliary stricture	Yes	1.006 (0.616–1.640)	0.982		
	No	Referent			
Benign biliary stricture	Yes	0.639 (0.212–1.926)	0.426		
	No	Referent			
CBD diameter	≤ 6 mm	0.874 (0.415–1.839)	0.723		
	> 6 mm	Referent			
Periampullary diverticulum	Yes	1.813 (1.061–3.098)	0.030	1.485 (0.784–2.815)	0.225
	No	Referent		Referent	
Impacted stone in the ampulla of Vater	Yes	1.147 (0.718–1.833)	0.567		
	No	Referent			
Surgically altered anatomy	Yes	2.069 (0.993–4.311)	0.052	2.374 (1.020–5.526)	0.045
	No	Referent			
Endoscopists	D + E	2.375 (1.267–4.453)	0.007	3.593 (1.746–7.394)	0.001
	B + C	1.317 (0.779–2.226)	0.303	1.556 (0.830–2.917)	0.168
	A	Referent		Referent	
Bleeding during NKP	Yes	18.18 (8.559–38.62)	< 0.001	21.18 (9.614–46.68)	< 0.001
	No	Referent		Referent	
P-duct stent before NKP	Yes	0.783 (0.347–1.768)	0.556		
	No	Referent			
Early pre-cut	Yes	0.832 (0.480–1.444)	0.514		
	No	Referent			

*CBD* common bile duct, *NKP* needle-knife pre-cut papillotomy, *OR* odds ratio, *CI* confidence interval, *P-duct* pancreatic duct.

## Discussion

NKP is an important and indispensable procedure for ERCP endoscopists as it is a promising option for deep bile duct cannulation in cases wherein conventional cannulation methods fail^5,12^. As NKP is difficult and potentially dangerous, especially when performed by inexperienced endoscopists, it is important to understand the predictors of initial NKP failure to facilitate quality improvement. To our knowledge, this is the first study to report the predictors of initial NKP failure. Our results indicated that technical factors, such as bleeding during NKP and the endoscopists’ experience, were the main factors associated with initial NKP failure. Surgically altered anatomy was the only patient factor associated with initial NKP failure on multivariate analysis.

Bleeding as a complication of needle knife pre-cut sphincterotomy was 3.03% in a systematic review of 7 randomized clinical trials (1 study with NKF or NKP, 2 with NKF, and the remaining 4 with NKP)^28^. Although in most cases, bleeding induced by NKP is reportedly mild and does not require blood transfusion, bleeding during NKP is a strong predictor of initial NKP failure in this study^29^. The key to the success of NKP is to identify the exposed CBD. However, when bleeding occurs and the endoscopic view is blocked, NKP usually results in failure. Kim et al. reported that 13% of the initial NKP failure reported in 69 patients were attributed to bleeding during the procedure^26^. In the present study, the incidence of bleeding during NKP was 12.1% in the entire population, but in the NKP-failure group, this rate increased to 38.9%. In contrast, the bleeding rate was only 3.4% in the NKP-success group. Therefore, during NKP, we recommend to control bleeding before it blocks the endoscopic field due to continuous bleeding as this may increase the success rate of NKP. According to our experience, due to the small caliber of most bleeding vessels, a needle knife (forced coagulation 30 W) can be used to stop the bleeding first. If the bleeding was relatively brisk or the needle knife was difficult to stop bleeding, we performed thermal coagulation using a heat probe (20 J) or hot biopsy forceps (soft coagulation 80 W).

Endoscopists’ experience is another important factor in initial NKP failure^30^. Like ERCP, it is difficult for beginners to master the skills required for NKP. Inexperienced endoscopists, for example, those performing not more than one sphincterotomy per week, had an initial NKP success rate of only 52%, but the complication rate could be as high as 24%^31^. Several studies have reported a learning curve involved in mastering the NKP technology; however, NKP was performed by a single endoscopist in their studies^2,32,33^. Unlike the above studies, the NKP procedures in this study were performed by five endoscopists. Each endoscopist might have different innate abilities and variations in learning style; hence, the number of cases of NKP treated by them would affect the success rate of the procedure. Our results further confirm that even among different endoscopists, mastering NKP involves a learning curve.

The surgically altered anatomy is an obstacle to the success of ERCP because it requires complete understanding of the reconstructed anatomy as well as in-depth knowledge regarding endoscopic intubation techniques or devices^6,34^. The direction of the biliary duct in patients with B-II gastrectomy and Roux-en-Y anastomosis is inferior, which is completely different from that in the general population^35,36^. Besides, an end-view endoscope rather than a side-view endoscope may be frequently used in the procedure^34^. As a result, many ERCP endoscopists may not have enough experience to be familiar with the correct bile duct axis, leading to the failure of NKP.

P-duct stents are increasingly used to reduce the risk or severity of post-ERCP pancreatitis, particularly before NKP or if cannulation is difficult^37^. Theoretically, the use of a P-duct stent as a guide for pre-cut may facilitate the performance of NKP^4^. However, due to a lack of studies, it is not clear whether the P-duct stent used for pre-cut will increase the success rate of NKP. Kubota et al. reported that NKF over a P-duct stent significantly increased the success rate of deep bile duct cannulation to 96.9% (95/98) compared with that of NKP without a pancreatic stent (86.1% [31/36], p = 0.0189)^27^. It was uncertain whether the higher success rate was attributed to NKF or the placement of P-duct stent, or both. In the present study, a P-duct stent before pre-cut did not affect the success rate of NKP. This might be due to the fact that only 10% of the study cohort received a P-duct stent, resulting in an insignificant result. Further randomized controlled studies are needed to clarify this issue.

There are several limitations to our study. First, this was a retrospective study from a single center. There were no uniform criteria for the definition of difficult cannulation. However, owing to the infeasibility of a prospective study and lack of similar studies, this study is valuable because of its large sample size. Furthermore, this study included five endoscopists with varying experiences in the NKP technique, and not a single operator.

In conclusion, NKP is a highly technique-sensitive procedure, as technique factors rather than patient factors are the major factors responsible for its initial failure. Therefore, NKP may only be reserved for experienced ERCP endoscopists.

## Data Availability

Deidentified individual participant data are available and will be provided on reasonable request to the corresponding author. The study protocol and analytic codes are also available.
